# Exogenous Calcium Alleviates Nocturnal Chilling-Induced Feedback Inhibition of Photosynthesis by Improving Sink Demand in Peanut (*Arachis hypogaea*)

**DOI:** 10.3389/fpls.2020.607029

**Published:** 2020-12-21

**Authors:** Di Wu, Yifei Liu, Jiayin Pang, Jean Wan Hong Yong, Yinglong Chen, Chunming Bai, Xiaori Han, Xinyue Liu, Zhiyu Sun, Siwei Zhang, Jing Sheng, Tianlai Li, Kadambot H.M. Siddique, Hans Lambers

**Affiliations:** ^1^College of Land and Environment, National Key Engineering Laboratory for Efficient Utilization of Soil and Fertilizer Resources, Northeast China Plant Nutrition and Fertilization Scientific Observation and Research Center for Ministry of Agriculture and Rural Affairs, Key Laboratory of Protected Horticulture of Education Ministry and Liaoning Province, Shenyang Agricultural University, Shenyang, China; ^2^The UWA Institute of Agriculture, The University of Western Australia, Perth, WA, Australia; ^3^School of Biological Sciences, The University of Western Australia, Perth, WA, Australia; ^4^School of Agriculture and Environment, The University of Western Australia, Perth, WA, Australia; ^5^Department of Biosystems and Technology, Swedish University of Agricultural Sciences, Alnarp, Sweden; ^6^Liaoning Academy of Agricultural Sciences, Shenyang, China; ^7^Jiangsu Academy of Agricultural Sciences, Nanjing, China; ^8^College of Resources and Environmental Sciences, Key Laboratory of Plant-Soil Interactions, Ministry of Education, National Academy of Agriculture Green Development, China Agricultural University, Beijing, China

**Keywords:** low night temperature, growth, calcium, photosynthesis, peanut

## Abstract

*Arachis hypogaea* (peanut) is a globally important oilseed crop with high nutritional value. However, upon exposure to overnight chilling stress, it shows poor growth and seedling necrosis in many cultivation areas worldwide. Calcium (Ca^2+^) enhances chilling resistance in various plant species. We undertook a pot experiment to investigate the effects of exogenous Ca^2+^ and a calmodulin (CaM) inhibitor on growth and photosynthetic characteristics of peanut exposed to low night temperature (LNT) stress following warm sunny days. The LNT stress reduced growth, leaf extension, biomass accumulation, gas exchange rates, and photosynthetic electron transport rates. Following LNT stress, we observed larger starch grains and a concomitant increase in nonstructural carbohydrates and hydrogen peroxide (H_2_O_2_) concentrations. The LNT stress further induced photoinhibition and caused structural damage to the chloroplast grana. Exogenous Ca^2+^ enhanced plant growth following LNT stress, possibly by allowing continued export of carbohydrates from leaves. Foliar Ca^2+^ likely alleviated the nocturnal chilling-dependent feedback limitation on photosynthesis in the daytime by increasing sink demand. The foliar Ca^2+^ pretreatment protected the photosystems from photoinhibition by facilitating cyclic electron flow (CEF) and decreasing the proton gradient (*Δ*pH) across thylakoid membranes during LNT stress. Foliar application of a CaM inhibitor increased the negative impact of LNT stress on photosynthetic processes, confirming that Ca^2+^–CaM played an important role in alleviating photosynthetic inhibition due to the overnight chilling-dependent feedback.

## Introduction

*Arachis hypogaea*, peanut or groundnut, is a grain legume crop with high nutritional value that is primarily grown in tropical and subtropical regions (annual production ∼46 million tons). It originates from tropical South America and provides a vital global source of vegetable oil and protein (Prasad et al., 2003; Bertioli et al., 2016; Lambers et al., 2020). Temperature is critical for peanut growth. Low, but non-freezing (0–12°C) temperature stress, particularly overnight chilling, is a major factor limiting peanut growth, which restricts its production areas (Bagnall et al., 1988; Wan, 2003; Liu et al., 2013; Song et al., 2020). Low-temperature extremes impose variable stresses on plant growth, and the chilling/low-temperature episodes in both the dark and the light may range from several hours to days (Allen and Ort, 2001).

Photosynthesis, a pivotal growth process, is sensitive to low-temperature stress. Preceding warmer ambient temperature and/or following high light exposure further intensifies the chilling-induced negative effects on photosynthetic processes (Powles et al., 1983; Liu et al., 2013; Zhang et al., 2014; Liu, 2020; Song et al., 2020). In peanut cultivation regions, especially those in northern China, severe low-temperature stress often occurs at night, followed by warm sunny days with high light intensity. The effects of nocturnal chilling stress (0–12°C) on the photosynthetic machinery have been assessed in several species with a tropical/subtropical origin, including coffee (*Coffea arabica*; Guo and Cao, 2004; Bauer et al., 2006), tomato (*Solanum lycopersicum*; Liu et al., 2012), soybean (*Glycine max*; Van Heerden et al., 2004), avocado (*Persea Americana*; Whiley, 1999), and mango (*Mangifera indica*; Nir et al., 1997; Allen et al., 2000). Little attention has been given to the physiological responses to peanut overnight chilling stress (Liu et al., 2013; Song et al., 2020). In regions prone to nocturnal chilling, the peanut is at risk of variable foliar curling and necrosis (Bagnall et al., 1988; Wan, 2003; Liu et al., 2013). With global climate change associated with the increasing frequency of extreme weather events, such as low night temperature (LNT), nocturnal/overnight chilling stress, and frost attacks in recent years, peanut production in temperate climate zones is facing new challenges (Cramer et al., 2018; Maxwell et al., 2019).

“Chemical priming” or the pretreatment of plants with selected chemical compounds can stimulate plant physiological mechanisms to cope with biotic or abiotic stresses (Beckers and Conrath, 2007; Savvides et al., 2016). Several approaches have been tested to examine its efficacy in ameliorating the adverse effects of chilling stress on crops. Exogenous foliar calcium (Ca^2+^) application can alleviate leaf damage and growth inhibition during chilling stress. Pretreatment of exogenous Ca^2+^ improved acclimation to chilling stress in low-temperature sensitive plant species, such as peanut (Liu et al., 2013; Song et al., 2020), wheat (*Triticum aestivum*; You et al., 2002), Chinese crab apple (*Malus hupehensis*; Li et al., 2017b), and tomato (Liu et al., 2012; Zhang et al., 2014), although the mechanism remains unclear (Liu, 2020). Ca^2+^, as an essential plant mineral nutrient, plays an important role in maintaining the stability of cell walls and membranes (Ali et al., 2003; Song et al., 2020). Ca^2+^ ions also serve as a ubiquitous second messenger in plant signal-transduction networks (Anil and Rao, 2001). Under abiotic stress, plants can initiate a series of physiological and biochemical processes by increasing the concentration of free Ca^2+^ in the cytosol and combining Ca^2+^ with calmodulin (CaM), thus playing an important role in the transmission, response, and acclimation of plants to multiple stresses (Kader and Lindberg, 2010). Ca^2+^ ions participate in a wide variety of environmental stresses, such as drought (La Verde et al., 2018), salt (Knight et al., 1997), low-temperature (Knight et al., 1996), oxidative stress (Price et al., 1994), and hypoxia (Subbaiah et al., 1994). Furthermore, Ca^2+^ is involved in regulating carbohydrate metabolism in the cytosol (Brauer et al., 1990), as well as increasing the translocation of photosynthetic carbohydrates to sinks (Joham, 1957; Navazio et al., 2020; Song et al., 2020).

Studies have demonstrated that foliar application of Ca^2+^ maintains leaf gas exchange and plant growth in peanut (Liu et al., 2013; Song et al., 2020), tomato (Zhang et al., 2014), and cucumber (*Cucumis sativus*; Zhang et al., 2012) exposed to LNT stress. Exogenous Ca^2+^ application sustains photosynthetic capacity by maintaining stomatal conductance (Chen et al., 2001), key enzyme activities in the Calvin-Benson-Bassham (CBB) cycle (You et al., 2002; Navazio et al., 2020), continued thylakoid electron transfer (Ai et al., 2006), and sustaining antioxidant capacity (Liu et al., 2015). Other studies have indicated that Ca^2+^ reduces the concentration of reactive oxygen species (ROS; Bhattacharjee, 2009; Liu et al., 2015), enhances cyclic electron flow (CEF; Zhang et al., 2014), and increases the xanthophyll cycle (Yang et al., 2013) during temperature stress. Applying Ca^2+^ improves cold resistance in tomato by increasing the concentration of soluble sugars, slowing down freezing, and enhancing the concentration of protoplasm in cells (Jiang et al., 2002; Liu et al., 2012).

In our previous study, foliar application of Ca^2+^ significantly enhanced peanut growth and photosynthesis under LNT stress and during its recovery under normal temperature (Liu et al., 2013; Song et al., 2020); however, the underlying physiological mechanism how exogenous Ca^2+^ alleviating inhibition of photosynthesis by nocturnal chilling in peanut remains poorly understood. Therefore, the present study examined the effects of exogenous Ca^2+^ and a CaM inhibitor, trifluoperazine (TFP) on photosynthetic reactions and growth in peanut exposed to long-term (days) and short-term (hours) LNT stress.

## Materials and Methods

### Plant Material and Experimental Design

The widely planted high-yielding peanut cultivar in China, Fenghua No. 2, was used in this study. Uniform peanut seeds were pre-germinated in a Petri dish for 36 h at 27°C and then planted in 32-cavity trays (one seed per cavity) for 7 days. Seedlings with average sizes were then transplanted into 150 pots (200 mm height, 260 mm diameter, 1 seedling per pot) filled with 4 kg of a standard horticultural substrate (Changchun Xihe Agro-technology Co. Ltd., Jilin, China). The pots were moved into an artificial climate chamber (Conviron, Winnipeg, Canada) with a day temperature of 25°C, night temperature of 20°C, and relative humidity (RH) of 60 ± 5%. All seedlings received a 12 h (from 6:00 to18:00) photoperiod at a photosynthetic photon flux density (PPFD) of 1,000 *μ*mol quanta·m^−2^·s^−1^ and CO_2_ concentration of 400 ± 5 μmol·mol^−1^. After 5 days of acclimation, 100 pots with uniform seedlings were selected and divided into four groups (25 pots per group) for the four treatments [LNT, LNT + Ca, LNT + TFP, and the control (CK); Table 1].

**Table 1:** Details of the four treatments used in the study.

Treatment	Day temperature	Night temperature	Foliar spray application (2 × daily) at 0, 5, and 10 days of LNT treatment
Control (CK)	25°C	20°C	Ultrapure water
LNT		8°C	Ultrapure water
LNT + Ca		8°C	15 mM Ca^2+^
LNT + TFP		8°C	5 mM TFP

The optimum concentration of exogenous Ca^2+^ (15 mM CaCl_2_) and CaM inhibitor (5 mM TFP) and the application technique were established in our previous experiments (Liu et al., 2013; Song et al., 2020). The seedling leaves were sprayed until dripping with ultrapure water. For the LNT + Ca and LNT + TFP treatments, 15 mM Ca^2+^ or 5 mM TFP, respectively, was evenly applied twice a day (at 8:00 and 16:00) on 3 days [0, 5, and 10 days of LNT treatment (DoL)]. In our previous experiments, we found that long-term LNT stress reduced leaf photosynthetic gas exchange significantly during the seedling stage. In our system, a duration of ≥1 DoL was defined as long-term LNT stress and <1 DoL was short-term LNT stress. This study assessed the effects of exogenous Ca^2+^ and a CaM inhibitor (TFP) on both long-term and short-term LNT stresses.

### Plant Sampling and Measurements

Three seedlings per treatment at 1, 6, and 11 DoL were selected for measurements of biomass, plant height, leaf area, leaf relative chlorophyll concentration, leaf gas exchange, and leaf hydrogen peroxide (H_2_O_2_) concentration. Chlorophyll was estimated on the third-youngest fully expanded leaf of the main stem with a chlorophyll meter (SPAD-502 Plus, Japan). Leaf gas exchange was measured on the same leaf using an open system (GFS-3000, Heinz Walz GmbH, Effeltrich, Germany) at 1, 6, and 11 DoL. During gas exchange measurements, the leaf cuvette temperature was set to 25°C and 60% RH. The CO_2_ concentration was maintained at 400 μmol·mol^−1^. An LED array provided a PPFD of 1,000 μmol quanta·m^−2^·s^−1^. The third-youngest fully expanded leaf was kept in the chamber, ensuring that the thermocouple touched it on the lower side. Leaf gas exchange parameters included net photosynthetic rate (Pn), stomatal conductance (g_s_), atmospheric CO_2_ concentration (C_a_), transpiration rate (Tr), intercellular CO_2_ concentration (C_i_), water-use efficiency (WUE=Pn/Tr), and leaf stomatal limitation ($Ls=1-C_{i}/C_{a}$). Leaf area was measured using an LI-3000C (LI-COR Biosciences, Lincoln NE, United States). After oven-drying at 105°C for 30 min and then 70°C to a constant weight, dry weights of leaves and whole plants were recorded. Leaf mass per unit leaf area (LMA) was calculated as LMA=leafdryweight/leaf area.

Hydrogen peroxide concentration was measured on the third-youngest fully expanded leaf of the main stem, as described by Li et al. (2017a). Briefly, finely ground leaves (60 mg fresh weight) were placed in a 2 ml microcentrifuge tube before adding 2 ml of 5% (w/v) TCA, and centrifuged 10,000 *g* for 10 min at 4°C. The supernatant (1 ml) was added to 0.1 ml of 20% (v/v) TiCl_4_ and 0.2 ml of concentrated ammonia. The mixture was centrifuged at 5,000 *g* for 10 min at 4°C. The pellet was dissolved in 3 ml of 1 M H_2_SO_4_ and the absorbance recorded at 410 nm.

At 6:00 AM on 1, 6, and 11 DoL, the third-youngest fully expanded leaves from six seedlings per treatment (pooled as three biological replicates per treatment) were ground to a powder after oven-drying at 105°C for 30 min and 70°C to constant weight for carbohydrate analysis. Soluble sugars were extracted from approximately 100 mg of the above leaf powder with 80% (v/v) ethanol at 85°C and quantified using the microtiter method (Hendrix, 1993). Pellets containing starch were oven-dried overnight at 60°C. Starch in the pellet was first gelatinized by adding 1 ml of 0.2 M KOH and incubated in a boiling water bath for 30 min (Rufty and Huber, 1983). After cooling, 0.2 ml of 1 M acetic acid was added, and the solution incubated with 2 ml acetate buffer (pH 4.6) containing amyloglucosidase (6 units, Roche, Basel, Switzerland) at 55°C for 1 h. The reaction was terminated in a boiling water bath, and the resulting supernatant analyzed for glucose (Song et al., 2020).

Chloroplast ultrastructure and chlorophyll fluorescence parameters were measured at 11 h of LNT treatment (HoL). Chloroplast ultrastructure was determined using methods previously reported (Strand et al., 1999). The third youngest fully expanded leaves were sliced and observed under a microscope at Centre for Microscopy, Characterization, and Analysis at Shenyang Agricultural University. The samples were fixed in 4% (v/v) glutaraldehyde, fixed after 2% (w/v) osmic acid, washed in 20 mM phosphate buffer, dehydrated by gradient ethanol, soaked in Epon812 resin, embedded, and polymerized. The resin was sliced (90 nm thickness) with a LEICA EM UC7 ultrathin slicer (Leica Microsystems, Wetzlar, Germany) and stained using uranyl acetate and lead citrate. The slices were observed and photographed by transmission electron microscopy (TEM; LSM 510; Carl-Zeiss AG, Oberkochen, Germany).

Chlorophyll fluorescence images were determined at 11 HoL with an imaging-pulse-amplitude-modulated (PAM) chlorophyll fluorometer (Heinz Walz, GmbH, Effeltrich, Germany) as described elsewhere (Li et al., 2014). Plants were fully dark-adjusted for >30 min at 11 HoL to measure the maximal photochemical efficiency of photosystem II $\left[\mathrm{Fv}/\mathrm{Fm}=\left(\mathrm{Fm}-\mathrm{Fo}\right)/\mathrm{Fm}\right]$ and the coefficient of non-photochemical quenching $\left(\mathrm{NPQ}=\mathrm{Fm}/\mathrm{Fm}\prime -1\right)$. Fluorescence images of leaves were obtained accordingly.

Measurements of rapid light curves (RLCs) of chlorophyll fluorescence parameters were determined at 11 HoL with Dual-PAM-100 measuring systems (Heinz Walz, GmbH, Effeltrich, Germany). The software Dual PAM v1.19 was used to control Dual-PAM-100 measuring systems to calculate the chlorophyll fluorescence and absorption changes simultaneously. Measurements were conducted using the software’s standard procedures and appropriate modifications based on our previous research (Shi et al., 2019; Song et al., 2020). The RLCs were determined after fully dark adjustment at 11 HoL (>30 min) at light intensities of 24, 32, 50, 108, 186, 286, 515, 773, 1,192, 1,469, and 1,823 μmol quanta·m^−2^·s^−1^. The exposure for each light intensity was 30 s and the saturation pulse was 1,000 μmol quanta·m^−2^·s^−1^ for 300 ms. All measurements were conducted at 25°C. The PSII parameters were measured using a Dual-PAM 100 device based on the saturation pulse method. The chlorophyll fluorescence parameters were calculated as: the actual quantum yield of PSII in the actinic light [AL; YII=Fm'−F/Fm], the quantum yield of non-regulatory energy dissipation $\left[Y\left(\mathrm{NO}\right)=F/\mathrm{Fm}\right]$, the regulatory quantum yield in PSII $\left[Y\left(\mathrm{NPQ}\right)=1-Y\left(\mathrm{II}\right)-Y\left(\mathrm{NO}\right)\right]$_,_ and the relative electron transfer rate of PSII $\left[\mathrm{ETR}\left(\mathrm{II}\right)=\mathrm{PAR}\times Y\left(\mathrm{II}\right)\times 0.84\times 0.5\right]$. The PSI parameters were measured using a Dual-PAM 100 device based on the P700 signal (absorption differences between 830 and 875 nm). The P700 parameters were calculated as: the actual quantum yield of PSI $\left[Y\left(I\right)=1-Y\left(\mathrm{NA}\right)-Y\left(\mathrm{ND}\right)\right]$, the quantum yield of non-photochemical energy dissipation due to donor-side limitation $\left[Y\left(\mathrm{ND}\right)=1-P700\mathrm{red}\right]$, the quantum yield of non-photochemical energy dissipation due to acceptor side limitation $\left[Y\left(\mathrm{NA}\right)=\left(\mathrm{Pm}-\mathrm{Pm}\prime \right)/\mathrm{Pm}\right]$, and the electron transfer rate of PSI $\left[\mathrm{ETR}\left(I\right)=\mathrm{PAR}\times Y\left(I\right)\times 0.84\times 0.5\right]$ (Schreiber and Klughammer, 2008). The CEF value $\left[\mathrm{CEF}=\mathrm{ETR}\left(I\right)-\mathrm{ETR}\left(\mathrm{II}\right)\right]$ and the ratio of the quantum yield of CEF to Y(II) $\left[Y\left(\mathrm{CEF}\right)/Y\left(\mathrm{II}\right)=\left(Y\left(I\right)-Y\left(\mathrm{II}\right)\right)/Y\left(\mathrm{II}\right)\right]$ were used to determine cyclic electron transfer (Yang et al., 2018).

A functionally intact photosynthetic apparatus was characterized by the slow decay of P515 signal after dark adaptation (high membrane integrity) and fast decay after illumination (high ATP-synthase activity; Schreiber and Klughammer, 2008; Zhang et al., 2014; Yang et al., 2018). In this study, the dual-beam 550–515 nm difference signal (electrochromic shift) was monitored simultaneously at 11 HoL using the P515/535 module of the Dual-PAM-100 (Heinz Walz, GmbH, Effeltrich, Germany). Balancing and calibrating of the P515 signal using the automated routine of the software Dual-PAM v1.19 occurred before each measurement (Schreiber and Klughammer, 2008; Suzuki et al., 2011). After 1 h of dark adjustment, P515 changes induced by saturating single turnover flashes were recorded to evaluate the integrity of the thylakoid membrane. After 10 min of pre-illumination at 630 *μ*mol quanta·m^−2^·s^−1^ and 4 min of dark adjustment, P515 changes induced by saturating single turnover flashes were recorded to evaluate ATP-synthase activity. Slow dark–light–dark induction transients of the 550–515 nm signals reflect changes in both membrane potential (electrochromic pigment absorbance shift) and zeaxanthin concentration. These transients were measured after 11 h of full dark adjustment. AL (630 μmol quanta m^−2^ s^−1^) was turned on after 30 s and off at 330 s. Based on analyzing light-off responses of the P515 signal, the membrane potential (*Δψ*) and proton gradient (ΔpH) components of the proton-motive force (pmf) were also assessed accordingly.

### Statistical Analyses

Statistical analyses were carried out using one-way ANOVA in SPSS 19.0 (Chicago, IL, United States). One-hundred uniform seedlings were included in this study and allocated to four groups (i.e., 25 seedlings per group). Three of the 25 seedlings per group were used for non-destructive measurements of leaf gas exchange, chlorophyll fluorescence, and P700 parameters. The remaining seedlings per treatment were selected for destructive sampling for seedling growth, TEM observations, and measurements of leaf area, biomass, and H_2_O_2_ and carbohydrate concentrations. The results are presented as mean values and SEs of three biological replicates. *Post hoc* LSD tests at *p* ≤ 0.05 were performed to determine differences among treatments. Significant differences are indicated as ^*^p ≤ 0.05 among treatments. All graphs were plotted using Origin 8.0 and Excel 2016 software.

## Results

### Long-Term LNT Stress

#### Plant Growth

**Figure 1:**
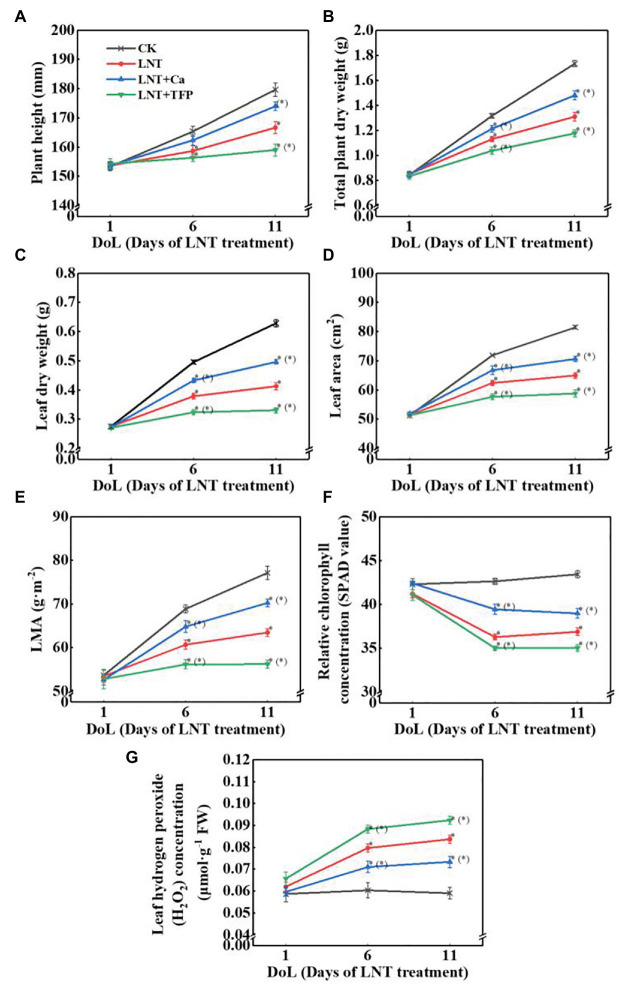
Effect of exogenous calcium (Ca^2+^) and a calmodulin inhibitor (TFP) on **(A)** plant height, **(B)** total plant dry weight (per plant including all organs), **(C)** leaf dry weight, **(D)** leaf area, **(E)** leaf mass per unit leaf area (LMA), **(F)** relative chlorophyll concentration (SPAD value), and **(G)** hydrogen peroxide (H_2_O_2_) concentration in peanut leaves under long-term low night temperature (LNT) stress [1, 6, and 11 days of LNT treatment (DoL)]. Values are means of three biological replicates ± SE (*n* = 3). ^*^indicate significant differences among treatments at *p* ≤ 0.05. Significant differences between the three treatments under LNT stress are shown in parentheses.

At 6 and 11 DoL, LNT decreased plant height, total plant dry weight, leaf dry weight, leaf area, LMA, and relative chlorophyll concentration in peanut, while the opposite was true for LNT + Ca. LNT + TFP further decreased these parameters at 6 and 11 DoL (Figures 1A–F). The LNT treatment had higher leaf H_2_O_2_ concentration than CK at 6 and 11 DoL; in contrast, LNT + Ca reduced it dramatically, and LNT + TFP increased it further (Figure 1G).

#### Concentrations of Soluble Sugars, Starch, and Total Nonstructural Carbohydrates

**Figure 2:**
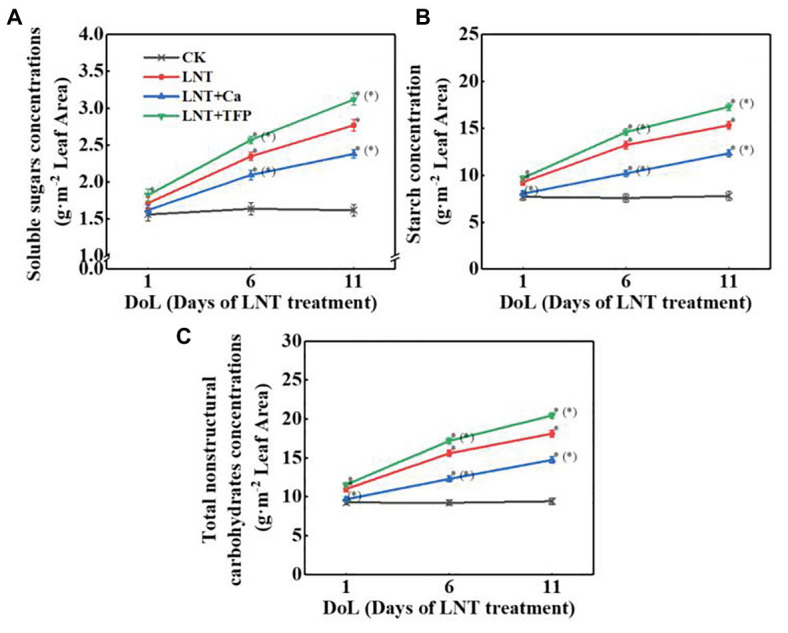
Effect of exogenous Ca^2+^ and a calmodulin inhibitor (TFP) on the concentrations of **(A)** soluble sugars, **(B)** starch, and **(C)** total nonstructural carbohydrates in peanut leaves under long-term LNT stress (1, 6, and 11 DoL). Values are means of three biological replicates ± SE (*n* = 3). ^*^indicates significant differences among treatments at *p* ≤ 0.05. Significant differences between the three treatments under LNT stress are shown in parentheses.

Low night temperature enhanced soluble sugar concentrations at 6 and 11 DoL, particularly and starch and total nonstructural carbohydrates at 1, 6, and 11 DoL. The reverse was true for LNT + Ca, relative to LNT. LNT + TFP increased the concentrations of soluble sugars, starch, and total nonstructural carbohydrates at 6 and 11 DoL (Figures 2A–C).

#### Leaf Gas Exchange

**Figure 3:**
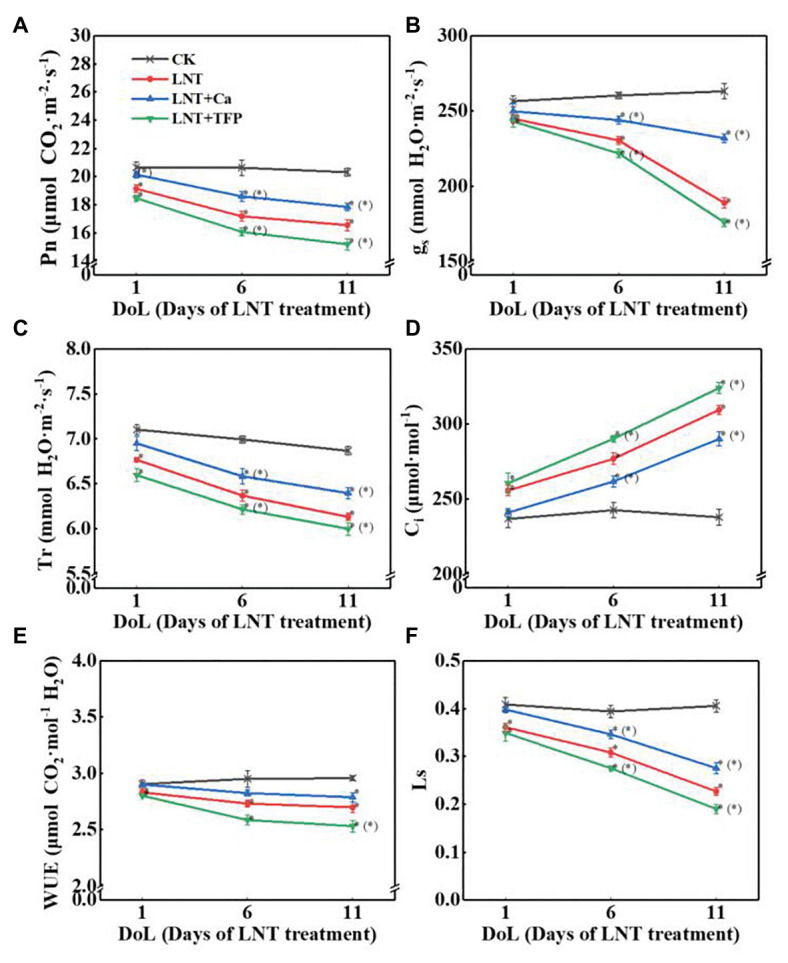
Effect of exogenous Ca^2+^ and a calmodulin inhibitor (TFP) on peanut gas exchange characteristics **(A)** net photosynthetic rate (Pn), **(B)** stomatal conductance (g_s_), **(C)** transpiration rate (Tr), **(D)** intercellular CO_2_ concentration (C_i_), **(E)** water-use efficiency (WUE), and **(F)** leaf stomatal limitation (Ls) in peanut leaves under long-term LNT stress (1, 6, and 11 DoL). Values are means of three biological replicates ± SE (*n* = 3). ^*^indicates significant differences among treatments at *p* ≤ 0.05. Significant differences between the three treatments under LNT stress are shown in parentheses.

Low night temperature decreased Pn, g_s_, Tr, WUE, and Ls and dramatically increased C_i_ at 1, 6, and 11 DoL. Compared with LNT, LNT + Ca increased dramatically Pn at 1, 6, and 11 DoL, increased g_s_, Tr, and Ls at 6 and 11 DoL, and markedly decreased C_i_ at 6 and 11 DoL. Conversely, LNT + TFP dramatically decreased Pn, g_s_, Tr, and Ls at 6 and 11 DoL, decreased WUE at 11 DoL, and increased C_i_ at 6 and 11 DoL (Figures 3A–E).

### Short-Term LNT Stress

#### Chloroplast Ultrastructure

**Figure 4:**
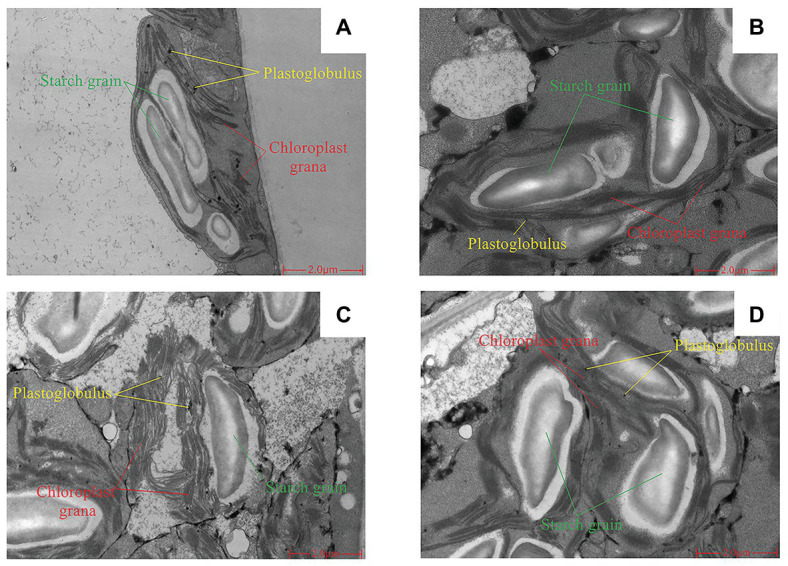
Effect of exogenous Ca^2+^ and a calmodulin inhibitor (TFP) on chloroplast ultrastructure of peanut leaves under short-term LNT stress (11 HoL). **(A)** CK; **(B)** LNT; **(C)** LNT + Ca, and **(D)** LNT + TFP.

Short-term LNT stress (11 HoL) damaged the chloroplast grana; LNT + Ca alleviated the damage, while LNT + TFP exacerbated the damage. Short-term LNT stress increased the size of starch grains and reduced the number of plastoglobules; LNT + Ca alleviated this effect, while LNT + TFP exacerbated it (Figures 4A–D).

#### Fv/Fm and NPQ

**Figure 5:**
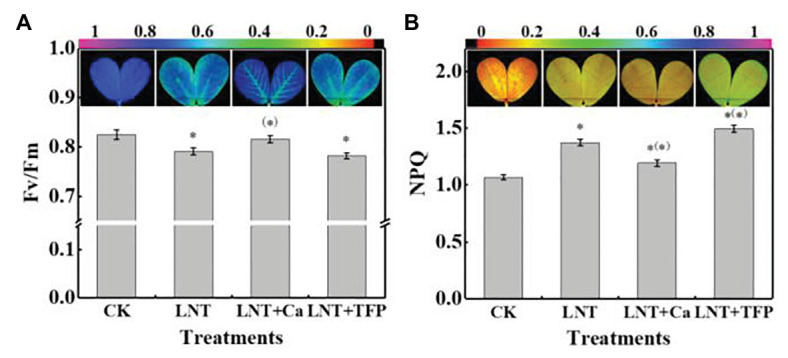
Effect of exogenous Ca^2+^ and a calmodulin inhibitor (TFP) on chlorophyll fluorescence parameters and images **(A)** maximum photochemical efficiency of PSII (Fv/Fm) and **(B)** non-photochemical quenching (NPQ) coefficient in peanut leaves under short-term LNT stress (11 HoL). Values are means of three biological replicates ± SE (*n* = 3). ^*^indicates significant differences among treatment at *p* ≤ 0.05. Significant differences between the three treatments under LNT stress are shown in parentheses.

The leaves in LNT treatment had lower Fv/Fm than CK, while LNT + Ca had similar values to CK (Figure 5A). The leaves in the LNT treatment had significantly higher NPQ than CK; LNT + Ca had lower NPQ and LNT + TFP had higher NPQ than LNT (Figure 5B).

#### Photosystems Activities

**Figure 6:**
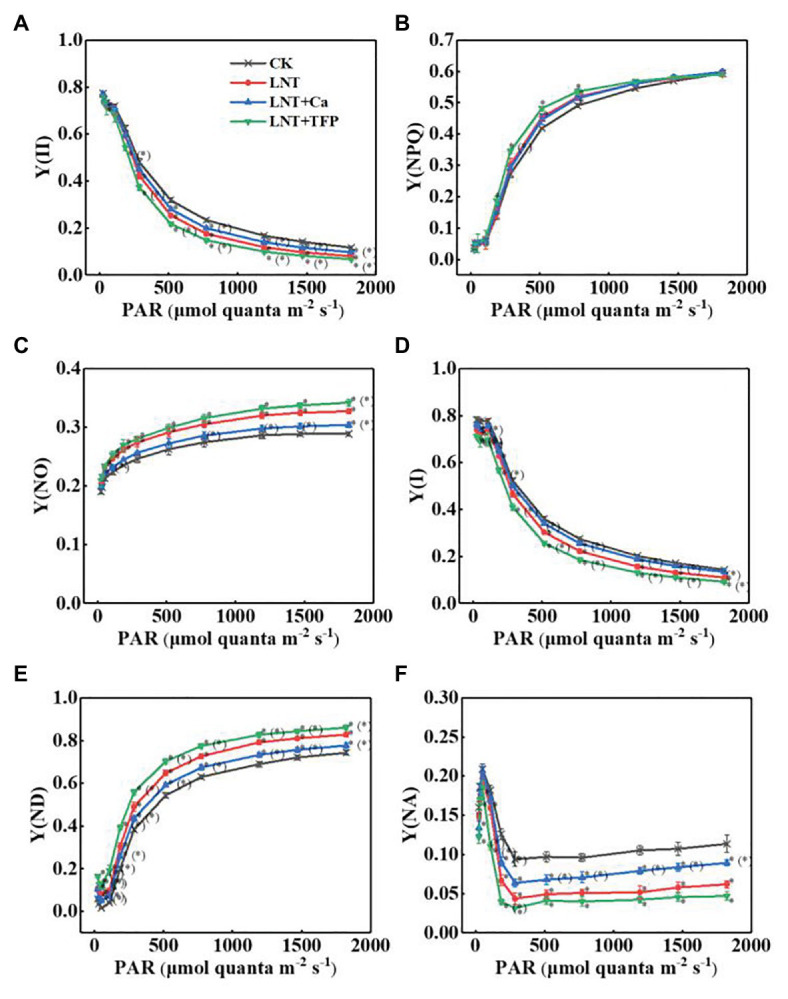
Effect of exogenous Ca^2+^ and a calmodulin inhibitor (TFP) on the rapid light curves (RLCs) of photosystems parameters **(A)** Y(II), **(B)** Y(NPQ), **(C)** Y(NO), **(D)** Y(I), **(E)** Y(ND), and **(F)** Y(NA) in peanut leaves under short-term LNT stress (11 HoL). Values are means of three biological replicates ± SE (*n* = 3). ^*^indicates significant differences among treatments at *p* ≤ 0.05. Significant differences between the three treatments under LNT stress are shown in parentheses.

The effective quantum yield of PSII photochemistry [Y(II)] decreased gradually with increasing light intensity in all treatments. The LNT treatment had significantly lower Y(II) than CK. LNT + Ca increased Y(II), while LNT + TFP reduced it further, relative to LNT (Figure 6A). The quantum yield of regulated energy dissipation in PSII [Y(NPQ)] increased rapidly with increasing light intensity in all treatments. The LNT treatment had significantly higher Y(NPQ) than CK before the light intensity reached 773 μmol quanta m^−2^ s^−1^. LNT + Ca decreased Y(NPQ), while LNT + TFP increased it further, relative to LNT (Figure 6B). In contrast, the quantum yield of non-regulated energy dissipation in PSII [Y(NO)] increased gradually with increasing light intensity. The LNT treatment had higher Y(NO) than CK. LNT + Ca decreased Y(NO), while LNT + TFP increased it, relative to LNT (Figure 6C).

The effective quantum yield of PSI photochemistry [Y(I)] followed the same trend as Y(II) (Figure 6D). The quantum yield of PSI non-photochemical energy dissipation due to the donor-side limitation [Y(ND)] increased gradually with increasing light intensity in all treatments. The LNT treatment had significantly higher Y(ND) than CK. LNT + Ca decreased Y(ND) while LNT + TFP increased it further, relative to LNT (Figure 6E). The quantum yield of PSI non-photochemical energy due to the acceptor-side limitation [Y(NA)] increased rapidly when initially exposed to light, before quickly declining and stabilizing in all treatments. The LNT treatment had significantly lower Y(NA) than CK. LNT + Ca enhanced Y(NA), while LNT + TFP decreased it further, relative to LNT (Figure 6F).

#### Photosynthetic Electron Transport

**Figure 7:**
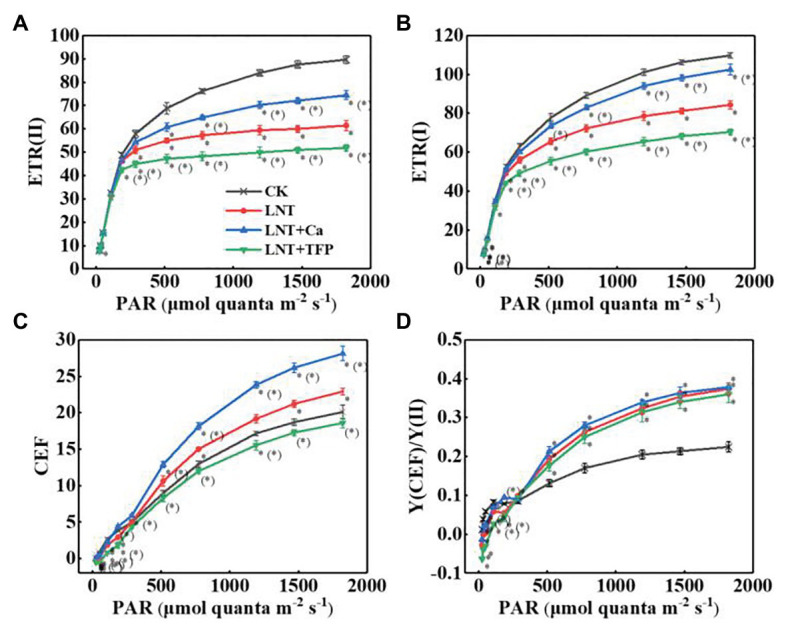
Effect of exogenous Ca^2+^ and a calmodulin inhibitor (TFP) on the RLCs of photosynthetic electron transport **(A)** ETR**(II)**, **(B)** ETR(I), **(C)** CEF, and **(D)** Y(CEF)/Y(II) in peanut leaves under short-term LNT stress (11 HoL). Values are means of three biological replicates ± SE (*n* = 3). ^*^indicates significant differences among treatment at *p* ≤ 0.05. Significant differences between the three treatments under LNT stress are shown in parentheses.

The electron transfer rate of PSII [ETR(II)] and PSI [ETR(I)] in leaves rapidly rose with increasing light intensity. The LNT treatment had significantly lower ETR(II) and ETR(I) than CK. LNT + Ca increased both ETR(II) and ETR(I), while LNT + TFP reduced them (Figures 7A,B). The CEF around PSI (CEF) increased with increasing light intensity in all treatments. The LNT treatment significantly increased CEF, relative to CK, and LNT + Ca stimulated it more than LNT, while LNT + TFP inhibited it from 186 μmol quanta m^−2^ s^−1^ onwards (Figure 7C). All LNT treatments had higher ratios of the quantum yield of CEF to Y(II) [Y(CEF)/Y(II)] than CK beyond 186 μmol quanta m^−2^ s^−1^ (Figure 7D).

#### Proton-Motive Force, Thylakoid Membrane Integrity, and ATP-Synthase Activity

**Figure 8:**
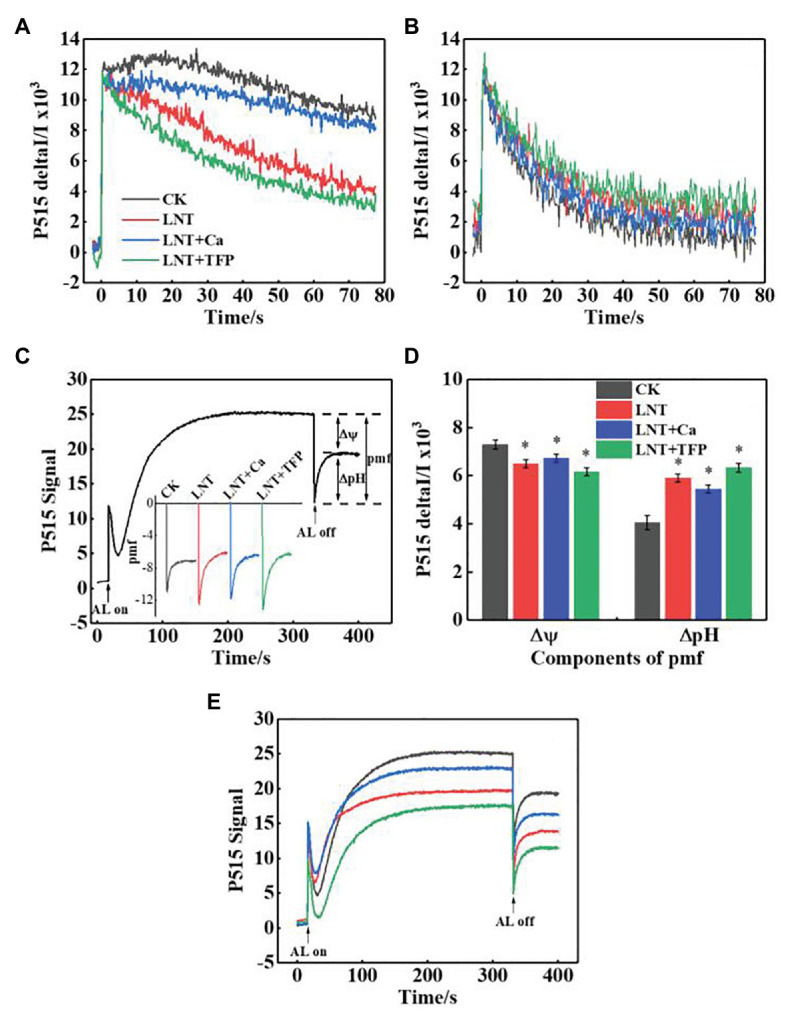
Effect of exogenous Ca^2+^ and a calmodulin inhibitor (TFP) on slow P515 induction transients of peanut leaves under short-term LNT stress (11 HoL). **(A)** Rapid kinetics of P515 induced by saturating single turnover flashes after dark acclimation for 1 h; **(B)** fast kinetics of P515 induced by saturating single turnover flashes after pre-illumination for 10 min at 1,000 *μ*mol photons·m^−2^·s^−1^ followed by 4 min darkness; **(C)** complete recording of light-on and light-off responses and enlarged display of light-off response to light quality with the indication of the estimated proton gradient (*Δ*pH) and membrane potential (Δ*ψ*) components of proton-motive force (pmf). Slow “dark–light–dark” induction transients of the 515 nm signal were measured. Actinic light (AL; 630 μmol·photons m^−2^ s^−1^) was turned on after 30 s and off after 330 s; **(D)** ∆pH and ∆ψ components of the pmf estimated from the curves of slow P515 kinetics; **(E)** changes in the P515 signal of slow dark–light–dark induction transients indicate the relative zeaxanthin concentration. Values are means of three biological replicates ± SE (*n* = 3). ^*^indicates significant differences among treatments at *p* ≤ 0.05. Significant differences between the three treatments under LNT stress are shown in parentheses.

The faster decay of the P515 signal after adjustment to darkness and the slower decay after irradiation to AL in the LNT treatments indicated that the thylakoid membrane integrity was impaired and inhibited ATP-synthase activity, relative to CK (Figures 8A,B). It also indicated that the rate of proton transfer from the lumen to the stroma *via* ATP-synthase was largely inhibited at 11 HoL. LNT + Ca pretreatment increased thylakoid membrane integrity and ATP-synthase activity while LNT + TFP decreased it.

The *Δψ* and ΔpH components of the proton-motive force (pmf) can be estimated by analyzing light-off responses of the P515 signal (Figure 8C). The difference between the signal of steady-state and the “dark baseline” reflects substantial Δψ. The “undershoot” below the “dark baseline” is considered a measure for the steady-state ΔpH. The LNT treatments had significantly lower Δψ than CK; LNT + Ca increased it further, and LNT + TFP slightly decreased it (Figure 8D). In contrast, the LNT treatments had significantly higher ΔpH than CK; LNT + Ca decreased it slightly, and LNT + TFP increased it slightly (Figure 8D). The relative extent of zeaxanthin formation can be judged from the increase in the “dark baseline” apparent after light-off (Figure 8E). The “dark baseline” of the LNT, LNT + Ca, and LNT + TFP treatments decreased significantly more than CK, indicating a decline in zeaxanthin concentration. Compared with LNT, LNT + Ca increased zeaxanthin concentration, while LNT + TFP decreased it.

## Discussion

### LNT Stress-Induced Feedback Inhibition of Photosynthesis Was Mainly Due to Limited Growth/Sink Demand

Nocturnal chilling stress significantly inhibited growth and leaf expansion (Figures 1A–E), which is consistent with earlier findings in tomato (Zhang et al., 2014; Lu et al., 2020) and melon (*Cucumis melo*; Hao et al., 2016). Other studies have demonstrated that peanut exhibited poor growth (associated with foliar necrosis and curling) when grown below 15°C (Wan, 2003; Liu et al., 2013). LNT stress generally reduces leaf area, stem diameter, and shoot and root dry matter accumulation (Solanke and Sharma, 2008; Dias et al., 2011; Hajihashemi et al., 2018). Leaf expansion rates change with the environmental temperature. Specifically, chilling stress reduces leaf expansion rates and leaf area in sorghum (*Sorghum bicolor*), maize, and sunflower (*Helianthus annuus*; Tardieu et al., 1999). LNT stress can also reduce leaf growth, the concentration of photosynthetic pigments and shoot and root dry matter accumulation in tomato (Latef and He, 2011), melon (Hao et al., 2016), and peanut (Song et al., 2020).

We demonstrated that even a short-term (11 HoL) overnight chilling stress significantly increased soluble sugar, starch, and total nonstructural carbohydrate concentrations in leaves (Figures 2A–C). Our TEM results confirmed that the short-term (11 HoL) LNT stress severely damaged the chloroplast grana and expansion of starch grains in leaves, relative to CK (Figures 4A,B). The accumulation of major photoassimilates (soluble sugars and starch) in leaves is critical for balancing photosynthate production and sugar consumption for tissue growth and development. A coordinated mutual relationship exists among plant growth/sink utilization and photosynthesis, rather than a simple one-way dependence of growth on photosynthesis (Adams et al., 2013; Lambers and Oliveira, 2019). Carbohydrate synthesis occurs in photosynthesizing leaves (sources) to provide substrates for plant growth (e.g., leaf expansion, stem, and root development) and maintain non-photosynthetic plant tissues (sinks; Cohu et al., 2014; Lambers and Oliveira, 2019). Our findings suggest that nocturnal chilling stress directly inhibits peanut growth and nonstructural carbohydrate translocation from source to sink, resulting in a significant accumulation of nonstructural carbohydrates in photosynthetically active leaves (Figures 2, 4), which are consistent with studies on maize (Adams et al., 2013) and peanut (Song et al., 2020).

This study showed that the negative impact of LNT stress on peanut photosynthesis was due to reduced export of nonstructural carbohydrates, as we only exposed plants to nocturnal chilling stress (Figures 1–4). The imbalance between source and sink/growth can further exert feedback downregulation or inhibition of leaf photosynthesis *via* nonstructural carbohydrate accumulation in photosynthesizing leaves (Foyer, 1988; Koch, 1996; Paul and Foyer, 2001; Paul and Pellny, 2003). In particular, we demonstrated that significant accumulation of nonstructural carbohydrates in leaves, even in short-term (11 HoL) LNT-stressed plants impaired photosynthetic machinery, including photosystems activities, thylakoid electron transport, carbon fixation, chloroplast morphology, and photoinhibition (Figures 3–8). Our results suggest that in the early stage of short-term LNT stress (without light), the significant accumulation of nonstructural carbohydrates damaged thylakoid membranes (Figures 4–6). Consequently, thylakoid membrane disintegration might be related to over-reduction and damage of the photosynthetic electron transport chain after short-term or long-term LNT stress followed by warm sunny days (with light; Figures 5–8; Song et al., 2020). Our findings are consistent with other studies, which reported that insufficient sink activity and growth inhibition can lead to significant accumulation of nonstructural carbohydrates in leaves and severe photoinhibition (Urban and Alphonsout, 2007; Adams et al., 2013). Indeed, there is evidence that long-term chilling/cold stress can inhibit the activities of photosynthetic reaction centers, thus restricting the electron transport chain and carbon fixation (Kasuga et al., 2004; Baker, 2008; Lu et al., 2020; Song et al., 2020). Our results further demonstrated that even a short-term LNT stress could also result in the decreases of the thylakoid membranes integrity and ATPase activity (Figure 7) and the increase of Y(NO), which is the non-regulated energy loss in PSII – a high value of Y(NO) reflecting the inability of the plant to protect itself against damage by excess excitation (Figure 6). It is plausible that the PSII super-complex was photo-damaged after the short-term (11 HoL) LNT stress (Figures 5, 6). The impact of the short-term LNT (11 HoL) stress on the light-dependent reactions was mainly reflected by slower electron transfer of thylakoids (Figure 7), reduced ATP and NADPH formation, and inhibition of carbon assimilation (Figures 3, 8), leading to significant H_2_O_2_ accumulation (Figure 1G) and impaired photosynthetic apparatus (Figures 5, 6). We also found that short-term LNT (11 HoL) stress stimulated the operation of cyclic photosynthetic electron transport around PSI, consistent with findings in Scots pine (*Pinus sylvestris*; Ivanov et al., 2001), maize (Savitch et al., 2011; Zhang et al., 2014), and tomato (Lu et al., 2020) exposed to long-term chilling stress.

### Chemical Priming by Exogenous Ca^2+^ Restored Nocturnal Chilling-Dependent Feedback Inhibition of Photosynthesis Was Mainly Due to Improved Growth/Sink Demand

Exogenous Ca^2+^ reduced the accumulation of nonstructural carbohydrates and H_2_O_2_ in leaves (sources) when undergoing overnight chilling stress (Figures 1, 2, 4). There are pieces of evidence that exogenous Ca^2+^ can serve to maintain photosynthetic processes by improving chilling stress resilience (Brauer et al., 1990) in tomato (Zhang et al., 2014; Liu et al., 2015), wheat (You et al., 2002), Chinese crab apple (Li et al., 2017b), and peanut (Liu et al., 2013; Song et al., 2020). In particular, Ca^2+^ is a critical essential element for peanut – a calciphilous legume crop – and directly connected to plant growth processes and responses to phytohormones (Wan, 2003; White and Broadley, 2003; Thor, 2019); and Ca^2+^ is involved in regulating a series of cellular activities, including plant cell division and elongation, cytoplasmic flow, and photomorphogenesis (Kader and Lindberg, 2010). The key function of Ca^2+^ is to serve as an intracellular messenger involved in many physiological processes and signaling pathways, ranging from plant tissue development (Michard et al., 2011; Monshausen et al., 2011; Ortiz-Ramírez et al., 2017; Zhang et al., 2017) to environmental stress responses (Knight et al., 1996, 1997). Ca^2+^ is involved in the regulation of carbohydrate metabolism, which can directly contribute to the regulation of sucrose synthesis, such as the inhibition of cytosolic Fru1,6-bisPase, activation of sucrose-phosphate synthase, and turnover of inorganic pyrophosphate (Brauer et al., 1990; Eckardt, 2001; Lu et al., 2013). In particular, Ca^2+^ is an important component of several signal-transduction pathways including sugar-signaling and auxin-signaling (Ohto and Nakamura, 1995; Gounaris, 2001). Moreover, Ca^2+^ regulation has been implicated in phloem function (Eckardt, 2001). Our results demonstrated that exogenous Ca^2+^ indirectly relieved a further decline in g_s_ and Tr under LNT stress (Figure 3), consistent with previous studies in *Arabidopsis* (Dong et al., 2013), cotton (*Gossypium hirsutum*; Joham, 1957), tomato (Liu et al., 2015), and spinach (*Spinacea oleracea*; Brauer et al., 1990), where Ca^2+^ improved the synthesis, phloem loading, and export of photosynthetic carbohydrates (Joham, 1957; Eckardt, 2001; Lu et al., 2013).

Based on our analyses, leaf morphology (Figures 1A–E), analytical chemical profiling (Figures 1F, 2), ultrastructural observations by TEM (Figure 4), gas exchange (Figure 3), and photosynthetic apparatus activity assessment (Figures 5–8) demonstrated that the restored LNT-linked damage to the photosynthetic machinery by exogenous Ca^2+^ might be a consequence, rather than a cause, of enhanced growth (sink demand) and stimulated export of nonstructural carbohydrates in photosynthesizing leaves. The accumulation of nonstructural carbohydrates in leaves generally impairs chloroplast structure, thylakoid membranes, and the photosynthetic electron transport chain (Foyer, 1988; Pammenter et al., 1993; Paul and Foyer, 2001; Song et al., 2020). We observed similar LNT-linked damage to peanut chloroplast structure and thylakoid membranes (Figures 3–8). Interestingly, exogenous Ca^2+^ relieved LNT impairment to chloroplast structure, thylakoid membranes, and photosystems activities; Ca^2+^ can bind to extrinsic luminal protein PsbO and sustain the oxygen-evolving complex (OEC; Heredia and Rivas, 2003; Sasi et al., 2018). The high concentration of Ca^2+^ in the lumen of the thylakoid membrane would stabilize the OEC against photodamage during environmental stress (Takahashi and Murata, 2008). In the present study, exogenous Ca^2+^ priming reduced Y(NO) undergoing short-term LNT stress, whereas short-term LNT and LNT + TFP resulted in an increase of Y(NO) indirectly (Figure 6C). It is known that Ca^2+^ application affects the expression of LHC stress-related protein 3, which is crucial for the energy-dependent component of NPQ (Terashima et al., 2012). In addition, exogenous Ca^2+^ can enhance the activities of several key enzymes in the Calvin-Benson-Bassham cycle, improving CEF and the PSII reaction center activity (Terashima et al., 2012; Hochmal et al., 2015). Our data suggested that Ca^2+^ priming helped to reduce damage to the PSI acceptor-side of the short-term LNT-stressed leaves by inducing a rapid increase in the CEF rate, thereby protecting the PSI reaction center (Figures 6E, 7C,D). More research is needed to ascertain the molecular changes associated with short and long-term LNT stress and the associated CEF pathway (i.e., the PGR5/PGRL1‐ or NDH-dependent CEF pathway) during Ca^2+^ priming.

We found that exogenous application of a CaM inhibitor (TFP) caused further downregulation of leaf physiology and additional growth inhibition (Figures 1–3). The LNT + TFP treatment caused a significant increase of soluble sugar, starch, and total nonstructural carbohydrate concentrations, relative to LNT (Figure 2). TFP enters plant cells through the cell membrane and prevents the formation of a Ca^2+^–CaM complex, which is essential for the functional CaM-linked signaling pathways during abiotic stress (Hepler, 2005; Liu et al., 2013). The Ca^2+^–CaM complex may play an important role in facilitating Ca^2+^ signal transduction to alleviate nocturnal chilling-dependent feedback inhibition of photosynthesis under short-term and long-term LNT stress. More research is needed to unravel the specific molecular mechanism(s) underpinning the Ca^2+^–CaM complex formation and signaling events during LNT stress.

Taken together, we show that exogenous Ca^2+^ alleviated nocturnal chilling-dependent feedback inhibition of photosynthesis. The impairment of the photosynthetic apparatus was prevented by improving sink demand through the continued export of nonstructural carbohydrates during exogenous Ca^2+^ priming.

## Conclusion

Both short-term (11 HoL) and long-term (1, 6, and 11 DoL) LNT stress inhibited peanut growth, leaf nonstructural carbohydrates export, and photosynthetic processes. Even a short-term LNT stress altered photosystems activities, thylakoid electron transport, and chloroplast morphology by causing significant accumulation of nonstructural carbohydrates in leaves. Our findings demonstrate that exogenous Ca^2+^ alleviated LNT-dependent feedback inhibition of photosynthesis by improving sink demand and facilitating nonstructural carbohydrate export from chloroplasts. In addition, Ca^2+^ priming reduced damage to the foliar photosynthetic electron transport chain by stimulating CEF and reducing the *Δ*pH. The poorer growth performance of TFP-pretreated seedlings than LNT-stressed seedlings confirmed the role of Ca^2+^ in alleviating LNT stress. These observations confirm the involvement of CaM in this Ca^2+^ priming restorative effect against LNT stress.

## Data Availability Statement

The original contributions presented in the study are included in the article/Supplementary Material, further inquiries can be directed to the corresponding author.

## Author Contributions

YL, TL, and XH designed the experiment. DW, CB, and SZ conducted the experiment and collected data for preliminary analysis. YL, JP, XL, ZS, and JS further analyzed the data and prepared the manuscript. HL, JP, JY, YC, and KS revised the manuscript. All authors contributed to the article and approved the submitted version.

### Conflict of Interest

The authors declare that the research was conducted in the absence of any commercial or financial relationships that could be construed as a potential conflict of interest.

## Abbreviations

CK: Control
C_i_: Intercellular CO_2_ concentration
CEF: Cyclic electron flow
DAT: Days after temperature treatment
ETR(I): Relative electron transport rate in Photosystem I
ETR(II): Relative electron transport rate in Photosystem II
F: Fluorescence yield measured briefly before application of a saturation pulse
Fo: Minimal fluorescence yield of the dark-adapted sample with all PSII centers open
Fo’: Minimal fluorescence yield of the illuminated sample with all PSII centers open
Fm: Maximal fluorescence yield of the dark-adapted sample with all PSII centers closed
Fm′: Maximal fluorescence yield of the illuminated sample with all PSII centers closed
Fv/Fm: Maximal photochemistry efficiency in Photosystem II
FH2: Fenghua 2
g_s_: Stomatal conductance
LNT: Low night temperature stress
TFP: Trifluoperazine
PAR: Photosynthetically active radiation measured in μmol quanta·m^−2^·s^−1^
Pred: P700 reduction coefficient under light
NPQ: Non-photochemical quenching
Fv’/Fm′: Light-adapted maximum quantum yield of PSII
OEC: Oxygen-evolving complex
PQ: Plastoquinone
Pm: Maximal P700 signal
Pm′: Real-time P700 signal under light
Pn: Net photosynthetic rate
PSI: Photosystem I
PSII: Photosystem II
qP: Photochemical quenching coefficient
ROS: Reactive oxygen species
Tr: Transpiration rate
Y(II): *Φ*PSII – Actual quantum yield in PSII under light
Y(NO): Non-regulatory quantum yield in PSII under light
Y(NPQ): Regulatory quantum yield in PSII under light
Y(I): ΦPSI – Actual quantum yield in PSI under light
Y(ND): Quantum yield of PSI non-photochemical energy dissipation due to the donor-side limitation
Y(NA): Quantum yield of PSI non-photochemical energy dissipation due to the acceptor-side limitation
Y(CEF)/Y(II): Ratio of the quantum yield of CEF to Y(II)
